# Analytical detection of the bioactive molecules dopamine, thyroxine, hydrogen peroxide, and glucose using CsPbBr_3_ perovskite nanocrystals†

**DOI:** 10.1039/d4ra06576j

**Published:** 2024-10-15

**Authors:** Puthanveedu Divya, Kodompatta P. Arjunan, Maya Nair, John P. Rappai, Kulangara Sandeep

**Affiliations:** a Government Victoria College, Research Centre Under University of Calicut Palakkad 678001 India sandeepk@gvc.ac.in; b Government Arts and Science College Ollu r Thrissur 680306 India

## Abstract

Qualitative and quantitative detection of biologically important molecules such as dopamine, thyroxine, hydrogen peroxide, and glucose, using newer and cheaper technology is of paramount importance in biology and medicine. Anion exchange in lead halide perovskites, on account of its good emission yield, facilitates the sensing of these molecules by the naked eye using ultraviolet light. Simple chemistry is used to generate chloride ions from analyte molecules. Dopamine and thyroxine have an amine functional group, which forms an adduct with an equivalent amount of volatile hydrochloric acid to yield chloride ions in solution. The reducing nature of hydrogen peroxide and glucose is used to generate chloride ions through a reaction with sodium hypochlorite in stoichiometric amounts. The emission of CsPbBr_3_-coated paper/glass substrates shifts to the blue region in the presence of chloride ions. This helps in the detection of the above biologically important molecules up to parts per million (ppm) levels by employing fundamental chemistry aspects and well-known anion exchange in perovskite nanocrystals. The preparation of better and more efficient sensors, which are predominantly important in science and technology, can thus be achieved by developing the above novel, cost-effective alternative sensing method.

## Abstract 2

Qualitative and quantitative detection of biologically important molecules such as dopamine, thyroxine, hydrogen peroxide, and glucose, using CsPbBr_3_ nanocrystals coated paper/glass substrates are discussed.

## Graphical abstract

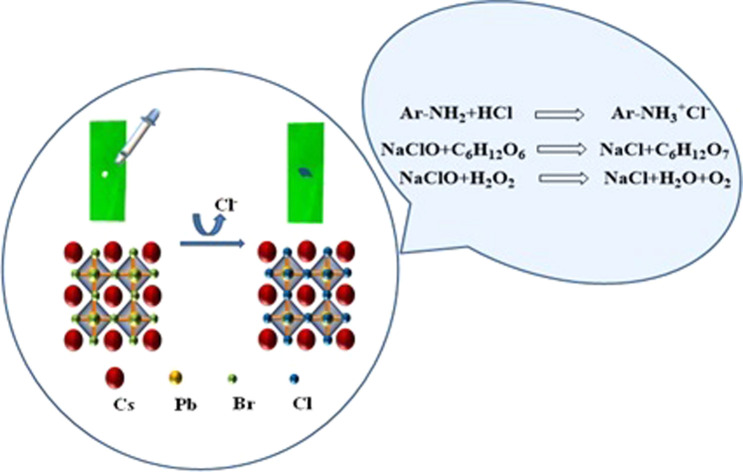

## Introduction

Regulation of a wide range of biomolecules determines the intricate and dynamic biological functioning of all living organisms. These vital molecules, such as dopamine, thyroxine, glucose, and hydrogen peroxide, are integral for the maintenance and precise functioning of biological processes. Among these molecules, the neurotransmitter dopamine is crucial for the ideal functioning of the renal, cardiovascular, hormonal, and mammalian central nervous systems.^1–7^ Dopamine is commonly known as the ‘happy hormone’ since it has a significant role in performing various tasks related to emotional health and pleasure. The lack of this neurotransmitter results in the evolution of Parkinson's disease, which affects the whole nervous system and parts of the human body controlled by it. Similarly, another important biomolecule is thyroxine, which is a primary hormone secreted by the thyroid gland, and it regulates metabolism in human beings. Thyroxine helps in the proper functioning of the heart, brain growth, digestion, and muscle operations.^8–10^ Hypothyroidism is a deficiency of thyroxine and is a common disease that affects 5% of the world's population.^11^ Hydrogen peroxide (H_2_O_2_) is another bio-significant molecule that plays a dual role in living organisms. H_2_O_2_ can act as a hazardous oxidant at higher concentrations. However, it functions as a cellular signaling molecule when its amount is at an optimum level. The enzymes peroxidase and catalase control the level of peroxide ions in the human body and help cells regulate oxidative stress. Chronic oxidative stress, due to reactive oxygen species formed by the imbalance in the concentration of peroxide ions, can end up in atherosclerosis. Glucose, yet another important biomolecule, plays a crucial role in animals as a food/pharmaceutical product. As a primary metabolite in many living organisms, glucose is considered one of the most important biomolecules.^12,13^ However, higher concentrations of glucose in the blood can result in diabetes mellitus, which again is an increasingly common health issue in human beings.

Successful sensing of these biomolecules has a huge impact on biomedical applications, and hence, highly accurate and sensitive sensing methods are essential. Multiple laboratory tests involving electrochemical, optical, thermometric, piezoelectric, and magneto-analytical techniques have been employed for the detection of these molecules.^14–19^ Amongst them, electrochemical sensors are widely used due to their better selectivity and reproducibility. However, all of them require expensive instrumentation and skilled technicians and yet face several issues regarding accuracy and reliability. Technical improvements for monitoring and sensing these molecules are a priority, and new simple methodologies must be developed taking into consideration the safety aspects in a scenario wherein the new technologies can be handled by the common people or laymen. Herein, we report a new methodology, where lead halide perovskite-coated paper strips/glass slides are used for the naked eye detection of these molecules in an ultraviolet chamber.

The manifold applications of lead halide perovskites in different areas of science and technology have captivated the scientific community because of their fascinating optoelectronic/redox properties. Their broad wavelength absorption and narrow emission with high photoluminescence quantum yield (PLQY) make them suitable for fluorescent sensor applications.^20–28^ Lead halide perovskites are now considered a promising candidate for solar cells, light-emitting diodes, and display applications.^29–46^ Post-synthetic band gap tuning by anion exchange is another important property of lead halide perovskites.^47–53^ Anion exchange of bromide ions from lead bromide perovskites increases the bandgap of the semiconductor, which causes a blue shift in the absorption/emission.^54–56^ Simultaneously, the exchange of bromide ions with fewer electro-negative iodide ions lowers the bandgap, resulting in a bathochromic shift in the emission/absorption. Herein, anion exchange of lead halide perovskites is used to sense these molecules. By sample pre-treatment, an equivalent amount of chloride ions is generated from these biomolecules, and they are detected using the perovskites-coated paper strips/glass slides. All inorganic CsPbBr_3_ are used as the lead halide perovskites due to their better stability.

## Results and discussion

**Fig. 1:**
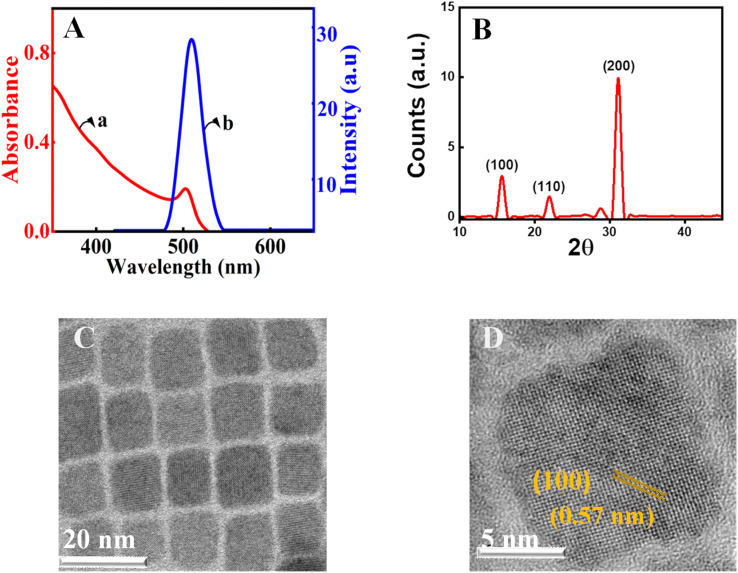
(A) Spectroscopic characterisation of CsPbBr_3_ perovskite nanocrystals: (a) emission spectrum (b) absorption spectrum of the synthesised nanocrystal. (B) X-ray diffraction pattern. (C) Low-resolution TEM and (D) HR-TEM images of CsPbBr_3_ perovskite crystals.

Herein, CsPbBr_3_ perovskite nanocrystals were produced by employing a well-known procedure with minor variations.^57^ Cs_2_CO_3_ was treated with oleic acid at 140 °C to yield an optically clear solution of cesium-oleate in a three-neck round bottom flask in an inert argon atmosphere. Parallelly, the capping agents, oleylamine and oleic acid, in octadecene were used to dissolve PbBr_2_ at 150 °C.^58^ Further, the CsPbBr_3_ nanocrystals were prepared by injecting the cesium-oleate lead oleylamine complex at 150 °C. Following the synthesis of CsPbBr_3_ perovskite nanocrystals, repeated precipitation and washing with acetone followed by centrifugation were used to purify the material. Details of the preparation and purification are given in the ESI†. The precipitated nanocrystals are redispersed in chloroform for further experiments. The synthesized nanocrystals are characterized by spectroscopic, electronic microscopy, and X-ray diffraction (XRD) techniques. The excitonic absorption, which is an indication of the band edge transition of the present CsPbBr_3_ nanocrystals in chloroform, is approximately 499 nm (Fig. 1A, trace a). All of the characteristic peaks are present in the absorption spectrum of CsPbBr_3_ perovskite nanocrystals.^59^

Further photoluminescence measurements are carried out to characterize the nanomaterials. In the emission study, the material is excited at 348 nm using a Xenon arc lamp and by keeping the emission and excitation slit widths at one nm. The emission maxima for the present nanocrystals were observed at 510 nm (trace b, Fig. 1A). The full width at half maximum of the emission was found to be 18 nm.^60–63^ This ensures that the synthesized perovskite nanocrystals are monodisperse. Furthermore, the crystalline nature of the manufactured perovskite is confirmed using powder XRD (Fig. 1B). The diffraction patterns at 2*θ* values of ∼15.0° and ∼31.0° are in agreement with the (100) and (200) crystallographic planes of the CsPbBr_3_ perovskites.^64^ The measured values are consistent with the standards and earlier known reports for the CsPbBr_3_ perovskite diffraction.^65^ Further, perovskite crystals are characterized with transmission electron microscopy (TEM). The observation from the low-resolution investigation finds the CsPbBr_3_ nanocrystals to be monodisperse and crystalline (Fig. 1C). The average size of nanocrystals is estimated with the help of a size-distribution histogram and is found to be 8.2 nm for the current perovskite nanocrystals (ESI†). The analysis of high-resolution TEM (HRTEM) using the Gatan digital micrograph software is used to obtain the *d*-spacing. In the present analysis, it is found to be 0.57 nm, which corresponds to the (100) plane of CsPbBr_3_ perovskite nanocrystals.

**Scheme 1:**
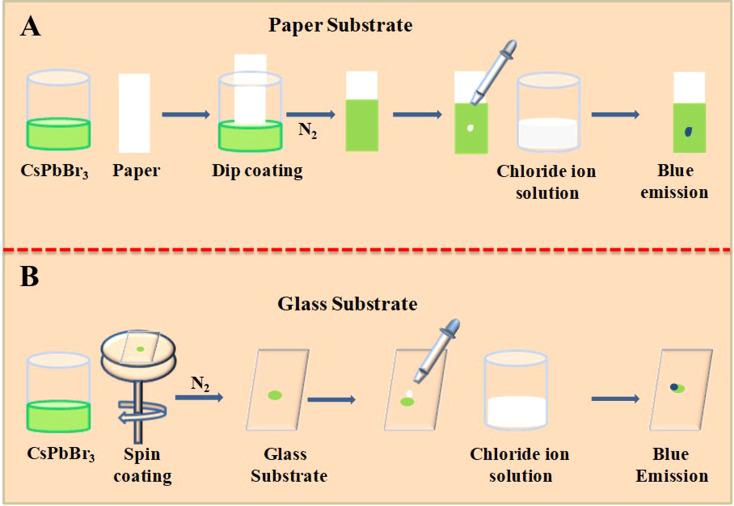
Schematic representation of coating the CsPbBr_3_ perovskite nanocrystals on a paper and glass substrate and its usage as a sensor for different analyte molecules.

**Fig. 2:**
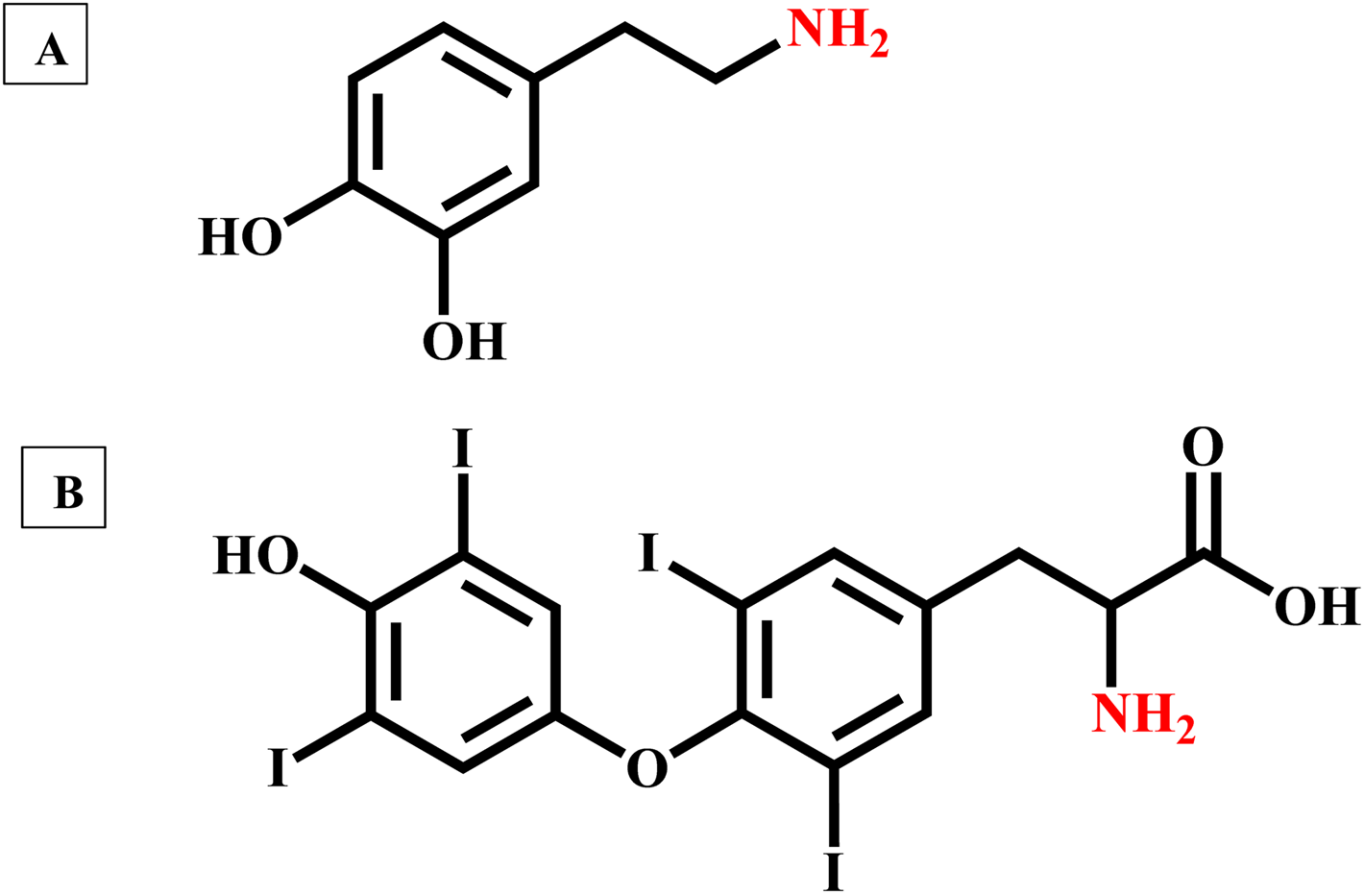
(A) Chemical structure of dopamine and (B) chemical structure of thyroxine.

Herein, we use the anion exchange of lead halide perovskites to detect biomolecules even though they do not have halide ions. These biologically important molecules are used to generate halide ions by simple chemical reactions. We need solid support for the perovskites and the anion exchange must take place in this substrate. In the present study, we have used paper/glass substrates. We have used the dip coating method to coat the CsPbBr_3_ nanocrystals on a Whatman 40 filter paper (Scheme 1A). After dipping the Whatman 40 filter paper in CsPbBr_3_ perovskites in chloroform, the paper is allowed to dry in a nitrogen atmosphere. Being volatile, chloroform quickly evaporates in the nitrogen flow. In an earlier work, we used similar paper substrates to detect fluoride, chloride, bromide, and iodide ions from water using the anion exchange reactions. Parallelly, a glass substrate coated with CsPbBr_3_ nanocrystals was also made, as marked in Scheme 1B. The CsPbBr_3_ perovskite in chloroform is spin-coated on a clean glass slide and dried under an inert atmosphere. Proper preparation and handling of the perovskite-coated substrates are important for the reproducibility and efficiency of sensing. Perovskite-coated glass/paper substrates enable naked-eye detection of these biologically important molecules in a UV chamber with high sensitivity. The fast anion exchange reactions and the high emission yield of lead halide perovskites ensure the sensing of the molecules presented in Fig. 2.

The neurotransmitter dopamine is a chemical messenger in our body and is derived from the amino acid tyrosine. Similarly, thyroxine, an amine hormone secreted by the thyroid gland, is important for the proper metabolism and functioning of the human body. In contrast to dopamine, thyroxine has three covalently bonded iodine atoms connected to the aromatic carbons. However, it is hard to generate iodide ions by nucleophilic substitution reactions. In the present context, we need to develop another method to generate halide ions from these two molecules. Even though dopamine and thyroxine serve distinct biological functions, they both have an amine functional group in their aromatic ring structure (Ar–NH_2_). It is well-known that the amines readily react with the mineral acids and form positively charged ammonium ions by combination reactions. Quaternary ammonium salts are known for their ionic nature. Herein, we have used hydrochloric acid (HCl) as the mineral acid, and upon reaction, these amine-containing biologically important molecules yield the corresponding quaternary ammonium chlorides (eqn (1)). HCl is a volatile acid, and the excess amount can be easily evaporated by gentle warming/keeping under infrared light. In the presence of concentrated HCl, perovskite-coated paper decomposes. Details are given in ESI.† Also, hydrochloric acid is commonly available and relatively cheaper. Upon evaporation of excess HCl, the quaternary salts of these biologically important molecules are obtained in the pure powder form.1
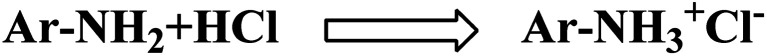


**Fig. 3:**
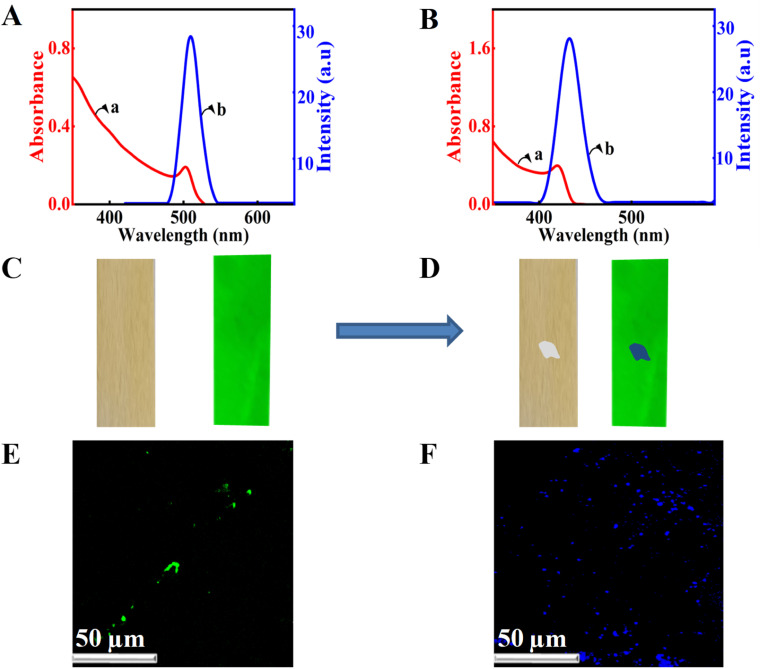
(A): (a) Absorption spectrum, (b) emission spectrum of the synthesised nanocrystal before and after anion exchange with chloride ions of dopamine hydrochloride, (B) paper strip coated with CsPbBr_3_ under visible and UV lamp, (C) optical microscopic images of CsPbBr_3_ NCs (green) before and (D) after the anion exchange reaction. The anion exchange resulted in the formation of blue-emitting CsPbCl_3_ NCs (D). Confocal fluorescence microscopic images of perovskite nanocrystals (E) before and (F) after anion exchange reaction under 407 nm laser excitation.

The chloride ions in the quaternary ammonium salt can be used for the anion exchange in lead halide perovskites, and spectroscopy can be used to detect amine-containing biomolecules. For this purpose, we have taken the absorption and emission spectra of the perovskite nanocrystals before and after treatment with the quaternary ammonium chloride (Fig. 3A and B). The presence of chloride ions resulted in blue-shifted absorption and emission (Fig. 3B). The blue shift in the emission of CsPbBr_3_ perovskites can be ascribed to the anion exchange reactions. In the presence of chloride ions, the bromide ions of CsPbBr_3_ are replaced by chloride ions, which results in the blue-shifted emission. Onsite detection using spectrometers is hard to perform; hence, we have developed another methodology for detecting these molecules. We used CsPbBr_3_-coated substrates and the solution of Ar–NH_3_Cl is dropped onto it and observed in a UV chamber with an excitation wavelength of 356 nm. Initially, we used perovskite-coated paper strips prepared as per Scheme 1. The normal emission of the CsPbBr_3_ perovskite-coated paper strip is green under UV illumination. The photographs of the CsPbBr_3_ coated paper strip under normal (left panel) and UV light (right panel) are presented in Fig. 3B. Interestingly, dropping a single drop of analyte solution using a capillary tube resulted in a blue shift in the emission of the perovskite-coated paper strip in the spotted area (Fig. 3D). Also, the changes are clear and observable with bare eyes. Additionally, we repeated the experiment with the CsPbBr_3_-coated glass substrate and observed the same results. Furthermore, confocal fluorescence microscopy was used to investigate the anion exchange reactions in the glass substrate (Fig. 3E and F). Before treatment with chloride ions, the perovskite-coated glass substrates showed green color particles (Fig. 3E) under the 407 nm laser excitation. Upon treatment with Ar–NH_3_Cl, the color of the emission was changed to blue under similar conditions (Fig. 3F). The detection of dopamine hydrochloride is presented in Fig. 3. Further, the experiment was repeated with different concentrations of these biologically important molecules to obtain the detection limit with the naked eye. With the present substrates, we could accurately monitor the sensing of dopamine up to 5 parts per million (ppm) using a UV chamber.

Using a similar method, we detected thyroxine hydrochloride, which was prepared by treating thyroxine with hydrochloric acid. The sample was dried perfectly under infrared radiation to obtain thyroxine hydrochloride. The sample was coated on a perovskite-coated paper substrate, and the shift in emission was observed in an ultraviolet chamber. Herein, we could detect thyroxine up to 163 ppm (ESI). All amine-containing molecules can be detected using this method. Good-quality substrates are essential for effective detection. The detection limit slightly varies with the quality of the CsPbBr_3_ perovskite-coated paper substrates. We repeated the experiments in the biological pH 7.4 using a phosphate buffer. We could detect the presence of these two biologically important molecules by anion exchange reactions. However, the decomposition of perovskites was found to be faster in the presence of a phosphate buffer.

In the above cases, combination reaction chemistry is used to generate chloride ions. Further, we have used redox chemical reactions to detect glucose and hydrogen peroxide. The proper detection of glucose is very important in cases of diabetes mellitus. Due to the presence of the aldehyde functional group, glucose shows a reducing nature. From the chemical formula (C_6_H_12_O_6_), it is clear that the molecule does not have any halide ions.2
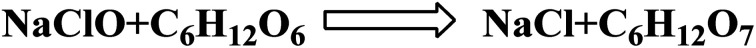


We used a common oxidizing agent, sodium hypochlorite (NaClO), to generate a halide ion. The chlorine in sodium hypochlorite is in a +1 oxidation state and cannot participate in the anion exchange reactions. However, the presence of reducing sugar in stoichiometric amounts can generate chloride ions (eqn (2)). By using the above perovskite-coated substrates, we could detect glucose up to 90 ppm. Details are provided in ESI.†

Similarly, hydrogen peroxide (H_2_O_2_), another biologically important molecule, reduces the Cl^+^ ions of sodium hypochlorite to Cl^−^ ions (eqn (3)). Inherently, hydrogen peroxide or hypo do not have chloride ions. However, after the reaction, it yields a chloride ion (eqn (3)). The detection limit of the peroxide ions was found to be 9 ppm. Details of the detections are given in the ESI.†3
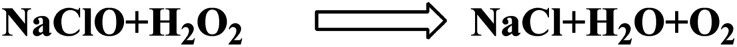


The field of sensing technologies has advanced significantly in recent years and has resulted in improvements in areas like industrial operations, environmental monitoring, and healthcare. However, the scope for developing methodologies that focus on fundamental chemistry aspects for developing better sensing technologies will certainly benefit humanity. Sensing using lead halide perovskites is cost-effective, and the tuneable band gap makes them the most suitable and reliable alternative to traditional sensing methods. The utilization of lead halide perovskites in sensing applications enhances the accuracy and efficiency of diagnosis, ranging from areas of healthcare to key industrial facets of product quality and safety. The detection of hydrogen peroxide, glucose, thyroxine, and dopamine with lead halide perovskites is a prime example of how nanotechnology can benefit and provide better solutions to varied issues and industrial applications. These advances enhance industrial operations, environmental monitoring, and healthcare by offering sensing platforms that are sensitive, accurate, and reasonably priced. The current progress in the study of lead halide perovskite points to a future in which sensing technologies will be essential in improving the general well-being of society.

## Conclusion

In summary, a cost-effective sensor for detecting biologically important molecules like hydrogen peroxide, glucose, thyroxine, and dopamine was prepared by coating CsPbBr_3_ perovskite nanocrystals on a Whatman 40 filter paper/glass slide. The combination and redox reactions therein made use of their halide precursor generation. The color changes in the emission that followed occurred because of an anion exchange reaction with perovskite nanocrystals and could be observed under a UV lamp in ppm level detection limits. The very high photoluminescent quantum yield of lead halide perovskite nanocrystals makes them a suitable candidate for the onsite detection of the above biologically important molecules with the help of a UV source without any instruments. Thus, this cost-effective detection of biologically important molecules with bare eyes under UV sources without sophisticated instruments provides a pathway to sensing biological molecules by applying simple chemistry and holds the key to creating a sustainable and bright future for sensing technology.

## Data availability

We used the following software programs to prepare the manuscript: Origin. Microsoft Office. Chemdraw.

## Conflicts of interest

The authors declare no competing financial interest.

## Supplementary Material

RA-014-D4RA06576J-s001
