# Supplementary material for: Analytical detection of the bioactive molecules dopamine, thyroxine, hydrogen peroxide, and glucose using CsPbBr3 perovskite nanocrystals
Source: RSC Adv. 2024 Oct 15;14(44):32648–54. doi: 10.1039/d4ra06576j (PMC11475663; doi:10.1039/d4ra06576j)
Supplement: RA-014-D4RA06576J-s001 [file RA-014-D4RA06576J-s001.pdf]

Supplementary Information (SI) for RSC Advances.
This journal is © The Royal Society of Chemistry 2024

Electronic Supplementary Information:

Analytical Detection of Bioactive Molecules Dopamine, Thyroxine, Hydrogen Peroxide, and Glucose using $CsPbBr_3$ Perovskite Nanocrystals

Puthanveedu Divya, Kodompatta P. Arjunan, Maya Nair, John P. Rappai, Kulangara Sandeep*

Department of Chemistry, Government Victoria College, Research center under University of Calicut, Palakkad, Kerala, India-678001

E-mail: sandeepk@gvc.ac.in

	Table of Contents	Page No.
1	Materials and methods	S2
2	Synthesis of $CsPbBr_3$ perovskite nanocrystals	S3
3	Characterization of $CsPbBr_3$ perovskite nanocrystals	S3
4	Calculation of crystal size of $CsPbBr_3$ perovskite nanocrystals from XRD	S4
5	Preparation of $CsPbBr_3$ coated paper/glass substrates	S4
6	Pretreatment Reactions	S4
7	Photographs of the emission changes by the action of HCl	S5
8	Photographs of the emission changes at the detection limit	S6
9	Photographs of the perovskite emission before and after treatment with pure biomolecule	S8

1. Materials and methods

Chemicals: Hydrogen peroxide (H_2O_2), glucose ($C_6H_{12}O_6$), dopamine ($C_8H_{11}NO_2$), thyroxine ($C_{15}H_{11}NO_4I_4$), sodium hypochlorite (NaClO) cesium carbonate (Cs_2CO_3, 99.99%), octadecene (98%), oleylamine (90%), and lead bromide ($PbBr_2$) are purchased from Sigma-Aldrich. Oleic acid (98%) is obtained from Alfa- Aesar. Chloroform (HPLC grade) and acetone are purchased from Merck chemicals. Hydrochloric acid (HCl) (99%) purchased from Nice chemicals. All chemicals are used as received without any further purification. All the solvents are used without any further purification.

Instrumental methods: The absorption spectra is recorded using a Shimadzu double beam spectrophotometer (Model UV-3600, UV-Vis-NIR), which is connected with three detectors (PMT, In GaAs, and PbS). A Horiba spectrometer (model JobinYvon SPEX-Fluorolog 3) is used for the emission analysis. To excite the samples, a 450W Xenon arc light is utilized. Double monochromators are utilized in emission and excitation chambers. The R928P side-on photomultiplier tube is used to detect the photons that are released from the sample. The sample's emission spectra were obtained by stimulating them at 340 nm while maintaining an emission slit width of 2 nm. Every steady-state absorption and emission measurement is performed at room temperature using a quartz cuvette (make: Starna) with a path length of one centimeter. For X-ray diffraction (XRD) examination, a PANalytical X'Pert Pro diffractometer is utilized, which has a Ni-filtered Cu-Kα line with a wavelength of 0.1540598 nm (2θ adjustable from 10° to 45o in 0.02^o increments). The sample is drop-casted onto a carbon-coated Cu grid for high resolution TEM (HRTEM) investigations, after which the solvent is allowed to evaporate. Specimens are imaged on an FEI TECNAI 30 G^2HRTEM and energy dispersive spectroscopic (EDS) analysis is also carried out using the same instrument with another detector (Si (Li)). Software called Gatan digital micrograph is used to perform the d-spacing study.

2. Synthesis of $CsPbBr_3$ perovskite nanocrystals

$CsPbBr_3$ perovskite nanocrystals are produced by employing a well-known procedure with some minor variations. For that purpose, one equivalence of Cs_2CO_3 is treated with two equivalence of oleic acid at 140°C, to yield an optically clear solution of cesium-oleate in a three neck round bottom flask, under an inert atmosphere of argon. In parallel, the capping agents oleylamine (1.15mmol, 1.5 ml) and oleic acid in octadecene (high boiling solvent, 5 ml) is used to dissolve $PbBr_2$ (0.19 mmol, 69.73 g) at 150°C. Further, the $CsPbBr_3$ nanocrystals are prepared by injecting the cesium-oleate lead oleylamine complex at 150°C. After the formation of $CsPbBr_3$ perovskite nanocrystals, they were purified by repeated precipitation and washing with acetone followed by the centrifugation. The centrifugation process removes the excess ligands and starting materials. After being purified, the nanocrystals are re-dispersed in HPLC-grade chloroform for further analysis.

3. Characterization of $CsPbBr_3$ perovskite nanocrystals

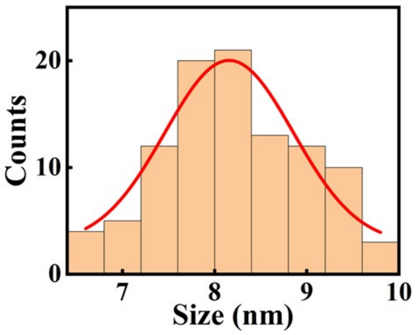

FigureS1: Characterization $CsPbBr_3$perovskitenanocrystals: (A) histogram of size from TEM analysis and (B) EDS spectrum.

4. Calculation of crystal size of $CsPbBr_3$ perovskite nanocrystals from XRD

According to schrerrer equation,

$$\textbf{Grain size (nm)} = \frac{\mathbf{K\,\lambda}}{\boldsymbol{\beta}\,\mathbf{cos\theta}}$$

K (Dimensionless shape factor) = 0.89

λ (wavelength of X-ray) = 0.154 nm

β (Full width at half maximum in radian) = 0.017

2θ = 31.1

By substituting these values, the grain size was found to be 8.36 nm, which is in accordance with the average crystal size calculated from TEM.

5. Preparation of $CsPbBr_3$ coated paper/glass substrates

Using the dip-coating technique, $CsPbBr_3$ perovskite nano crystals were coated on a Whatman 40 filter paper. For two minutes, $CsPbBr_3$ dispersion in chloroform was dipped onto Whatman 40 filter paper. This paper was also dried in argon air or atmosphere. In parallel, a glass substrate coated with $CsPbBr_3$ nanocrystals is also made with the help of a spin coater. The $CsPbBr_3$ perovskite in chloroform is spin-coated into a clean glass slide and dried under an inert atmosphere.

6. Pretreatment Reactions

Initially dopamine and thyroxine are converted to their quaternary ammonium salt by treating with stoichiometric amount of HCl. Approximately 0.5g of dopamine is treated with few drops of HCl

and the excess HCl removed by drying under an IR lamp. Later 0.095g of dopamine hydrochloride was dissolved in 0.5 ml distilled water to make one molar stock solution. It is then diluted to different concentrations by dilutions and it is tested by treating the standard solutions with perovskite coated paper/ glass substrate. Dopamine can be detected up to 0.025 mM range with bare eyes. Similarly, thyroxine was first converted to its quaternary ammonium salt by treating with HCl and it is detectable up to 0.25 mM. One molar stock solution of glucose is prepared by dissolving 0.09g of glucose in 0.5 ml water. Subsequently the glucose solution is treated with two drops of sodium hypochlorite solution. It is then gently warmed to fasten up the rate of redox reaction. Later it is diluted to different concentrations by dilutions and it is tested by treating the standard solutions with perovskite coated paper/ glass substrate. Glucose can be detected up to 0.05 mM range with bare eyes. Finally, 3% W/V hydrogen peroxide is used for the analysis. Similar to the glucose detection hydrogen peroxide is also treated with sodium hypochlorite solution and is converted to the detectable form. The solution is then diluted and optimized the minimum detection range. Hydrogen peroxide is detectable up to 0.075 mM.

7. Photographs of the emission changes by the action of concentrated HCl

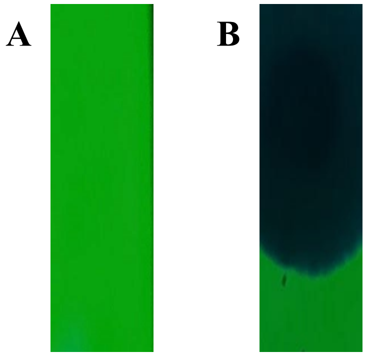

Figure S2: (A) Paper coated with $CsPbBr_3$, (B) $CsPbBr_3$ coated paper dropped with HCl

8. Photographs of the emission changes at the detection limit

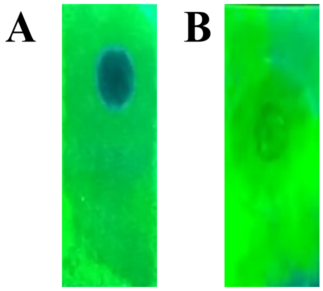

Figure S3: Photographs of $CsPbBr_3$ perovskite coated paper substrates viewed under a UV chamber of excitation wavelength 352 nm. (A) In the presence of 1 mM dopamine salt, (B) 0.025 mM dopamine salt.

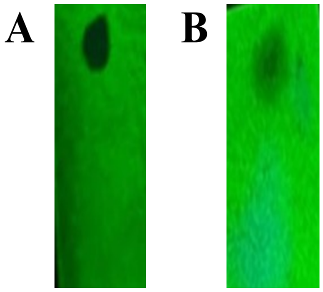

Figure S4: Photographs of $CsPbBr_3$ perovskite coated paper substrates viewed under a UV chamber of excitation wavelength 352 nm. (A) 10 mM thyroxine salt, (D) 0.25 mM thyroxine salt.

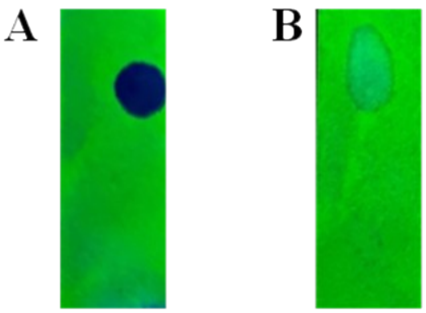

Figure S5: Photographs of $CsPbBr_3$ perovskite coated paper substrates viewed under a UV chamber of excitation wavelength 352 nm. (A) In the presence of 1 mM glucose, (B) 0.05 mM glucose.

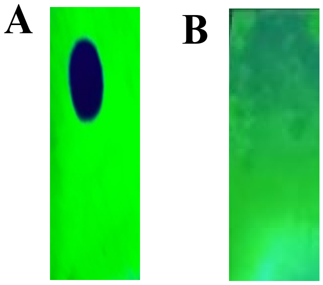

Figure S6: Photographs of $CsPbBr_3$ perovskite coated paper substrates viewed under a UV chamber of excitation wavelength 352 nm. (A) 1 mM Hydrogen peroxide, (D) 0.075 mm Hydrogen peroxide.

9. Photographs of the emission changes by the reaction with pure biomolecule.

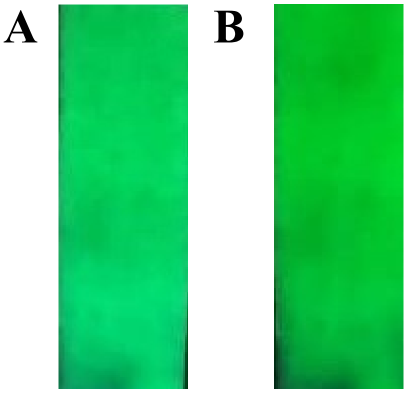

Figure S7: Photographs of $CsPbBr_3$ perovskite coated paper substrates viewed under a UV chamber of excitation wavelength 352 nm. (A) In the absence of dopamine, (B) In the presence of pure dopamine.

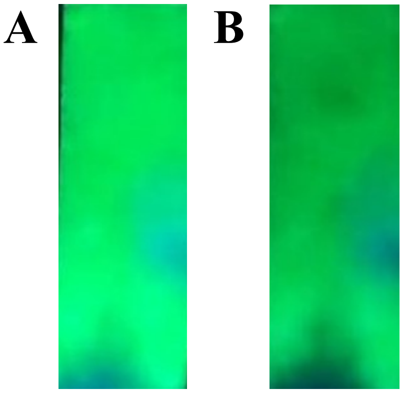

Figure S8: Photographs of $CsPbBr_3$ perovskite coated paper substrates viewed under a UV chamber of excitation wavelength 352 nm. (A) In the absence of thyroxine, (B) In the presence of pure thyroxine.

A B

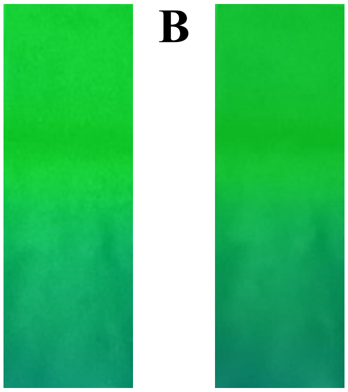

Figure S9: Photographs of $CsPbBr_3$ perovskite coated paper substrates viewed under a UV chamber of excitation wavelength 352 nm. (A) In the absence of glucose, (B) In the presence of pure glucose.

A B

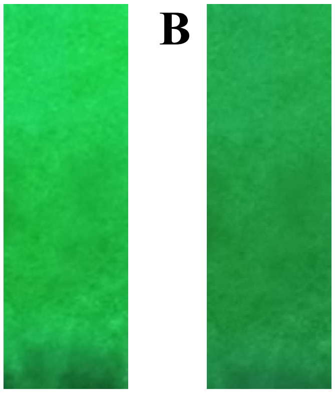

Figure S10: Photographs of $CsPbBr_3$ perovskite coated paper substrates viewed under a UV chamber of excitation wavelength 352 nm. (A) In the absence of Hydrogen peroxide, (B) In the presence of pure Hydrogen peroxide.
